# Air Pollution Health Risk Assessment (AP-HRA), Principles and Applications

**DOI:** 10.3390/ijerph18041935

**Published:** 2021-02-17

**Authors:** Tavoos Hassan Bhat, Guo Jiawen, Hooman Farzaneh

**Affiliations:** 1 Interdisciplinary Graduate School of Engineering Sciences, Kyushu University, Fukuoka 816-8580, Japan; tavoos.hassan.bhat.478@s.kyushu-u.ac.jp (T.H.B.); guo.jiawen.001@s.kyushu-u.ac.jp (G.J.); 2 China-UK Low Carbon College, Shanghai Jiao Tong University, Shanghai 200240, China

**Keywords:** air pollution exposure, health risk, air pollution aseessment tools, concentration-response functions

## Abstract

Air pollution is a major public health problem. A significant number of epidemiological studies have found a correlation between air quality and a wide variety of adverse health impacts emphasizing a considerable role of air pollution in the disease burden in the general population ranging from subclinical effects to premature death. Health risk assessment of air quality can play a key role at individual and global health promotion and disease prevention levels. The Air Pollution Health Risk Assessment (AP-HRA) forecasts the expected health effect of policies impacting air quality under the various policy, environmental and socio-economic circumstances, making it a key tool for guiding public policy decisions. This paper presents the concept of AP-HRA and offers an outline for the proper conducting of AP-HRA for different scenarios, explaining in broad terms how the health hazards of air emissions and their origins are measured and how air pollution-related impacts are quantified. In this paper, seven widely used AP-HRA tools will be deeply explored, taking into account their spatial resolution, technological factors, pollutants addressed, geographical scale, quantified health effects, method of classification, and operational characteristics. Finally, a comparative analysis of the proposed tools will be conducted, using the SWOT (strengths, weaknesses, opportunities, and threats) method.

## 1. Introduction

It is estimated that globally 8.9 million deaths happen due to air pollution exposure, resulting in 7.6% of the total yearly mortality and leading to 103.1 million healthy life years lost [1,2,3,4]. According to the World Health Organization (WHO), 4.2 Million lose their lives every year due to Ambient outdoor air pollution and 3.8 Million from indoor air pollution, mainly due to exposure to smoke from cookstoves and fuels [5]. Exposures to the particle material (PM) for the long term and short term have been indicated to increase mortality and reduce life expectancy [6,7,8,9]. It is assumed that by 2050 air pollution-related premature mortality could be double, and air pollution is perceived to be the most severe environmental health-related threats faced by the world [10]. Increases in mortality, morbidity, premature death, cardiovascular and respiratory diseases are some of the adverse effects due to air pollution exposure [11], Lung cancer [12], Adverse impact on the activity of the central nervous system resulting in cognitive impairment [13,14], and harmful effects on fetal development and pregnancy [15,16]. Air pollution, mostly particulate matter (PM), may have carcinogenic effects on humans [17,18,19]. Increased PM_10_ concentration by 10 μg/m^3^ has been indicated to increase non-accidental mortality [20,21,22]. Air pollution has been found to have an adverse economic impact worldwide, leading to the loss of GDP due to mortality and morbidity. With the increase in the GDP of the developing countries, the cost of air pollution has also been increasing. The economic impact is more evident in the urban areas [23,24,25,26,27,28]. Secondary pollutants such as ozone are also associated with respiratory, circulatory diseases, and mortalities [29,30], chronic respiratory diseases, and asthma [29]. Other studies have associated higher ozone concentrations with reproductive health [31], preterm birth [32], and cognitive disorders [33].

Since air pollution is now one of the most significant health hazards, there is a sufficient scientific basis to justify developing approaches to incorporate epidemiological assessment into the health-related risk. Although the idea of AP-HRA has been around since the 1950s, the health-care system worldwide has not adopted them as quickly. AP-HRAs can play a critical role at both individuals, community, and global health promotion and disease prevention levels.

According to the (WHO), “AP-HRAs estimate the health impact to be expected from measures that affect air quality, in different socioeconomic, environmental and policy circumstances. It is, therefore, an important tool for informing public policy decisions” [34]. It synthesizes information on exposures to air emissions, health impacts, and community risk used for regulatory decision-making and public participation [35].

AP-HRAs help to understand health benefits, which will be an outcome due to improved air quality [36,37] and has been used in many studies like the global burden of disease by WHO [3,38]. Over the last decade, they have evolved from more qualitative approaches to quantitative tools. HRA tools assess the health risks of the major pollutants such as oxides of sulfur (SOx) and oxides of nitrogen (NOx), ground-level ozone (O_3_), and particles (PM_2.5_) on the population which is exposed to these pollutants [39]. They relate the change in the level of the air pollutant concentration to the expected mortality rates due to ischemic heart diseases, stroke, lung cancer, and respiratory infections, using Concentration Response Functions (CRFs) [40]. Three main steps involved in developing the HRA tools include (1) population exposure assessment, (2) Health effect estimation related to air pollution, and (3) calculation of the uncertainty of the analysis [34].

The HRA tools can facilitate policy decision-making by evaluating the associated costs and health benefits of climate change mitigation actions. The urgency of bold and timely Low Emission Development Strategies (LEDS) coupled with the health, environmental, and economic opportunities has been argued in China and Mongolia [41,42]. These tools help raise public awareness regarding the adverse health impact of low air quality and finally connect governing authorities with scientific research throughout the regulatory process [43,44,45]. The HRA tools have been widely used in evaluating air quality policies in the United States [46] and the European Union [47]. Many countries have developed their own Nationally Appreciate Mitigation Action (NAMA) based on using the HRA tools, taking into account the different air pollution reduction scenarios. These studies range from local, national, regional, and global scales, which are reported in Table 1.

## 2. Methodological Approaches Used in the AP-HRAs

**Figure 1:**
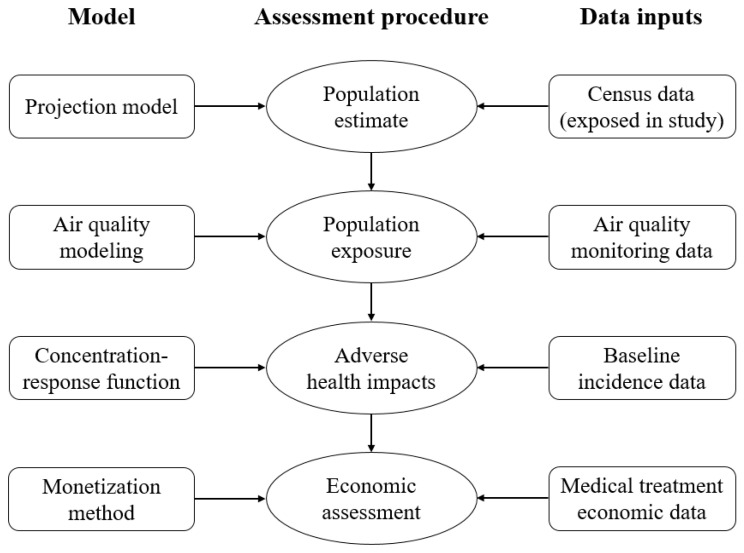
The flow diagram of Air Pollution Health Risk Assessment (AP-HRA) methods, typical models, and data inputs.

The health risk assessment for air pollutions contains the mathematical estimation and modeling of several processes, including population estimates, population exposure to pollutants, and adverse health impacts assessment through specific concentration-response functions [63]. In general, precise data are required, such as population data, air quality data, baseline mortality or disease rates, and risk estimation (change of the health effect related to the concentration change of air pollutants, which is referred to as coefficient, β) from epidemiological studies that quantify the association between health effects and exposure to air pollution. The flow diagram (see Figure 1) represents the methods, typical models, and data inputs of AP-HRA.

### 2.1. Population Estimates

The first stage of AP-HRA is to estimate the population exposed to air pollution once the temporal and spatial resolution in the study has been determined. Past and current data is accessible from some national census databases or the latest World Population Prospects published by the UN Department of Economic and Social Affairs [64]. In most cases, the health risk assessment is conducted for a particular socio-economic and environmental scope with some potential mitigation policies to be implemented. Therefore, the population data for the incoming few years achieved from population forecast models is usually required for the scenario setting.

### 2.2. Population Exposure to Air Pollution

The adverse health impacts are mainly derived from population exposure to contaminated air. Therefore, one core component of AP-HRA is the assessment of exposure to specific air pollutants for the target population, which is a comprehensive integral part of pollution concentration, the time-activity pattern of the population of interest (e.g., exposure period and level), the proportion of susceptible population and characteristics of pollutants (e.g., solubility and pattern of physiological contact). Most of the studies take the ambient concentration of air pollutants as a surrogate indicator for pollution exposure, as the measurement is conducted much more simply and conveniently [65]. Environmental agencies worldwide have set the air quality criteria to identify the concentration for those health-related pollutants [66]. Typically, the WHO air quality guidelines (2005) determined specified indicators of four main air pollutants, including PM_10_/PM_2.5_ (particles with diameter less than 10 μm or 2.5 μm), NO_2_, SO_2_, and O_3_, and proposed the interim targets and air quality guidelines (AQG) (See Table 2) [67]. The interim targets are intended for countries as incremental steps to move towards AQG, and the guidelines are selected based on concentration-response functions to suggest the concentration level that, if achieved, would contribute to significant benefits for the protection of public health.

Generally, modeling and monitoring are two major methods to estimate population exposure. Monitoring data can be directly used by collecting past and current air quality data near the monitoring sites. At the same time, modeling measurements can be combined with advanced monitoring technologies to facilitate: (i) simulation of air quality in different geographical areas, using specific socioeconomic or environmental conditions; and (ii) prediction of changes in exposure, taking into account the future policy implementations [68,69,70].

Recent analytical methodologies that have been commonly adopted in estimating the population exposure to air pollution can be classified as follows:

1. The Global Model of Ambient Particulates model (GMAPS) which was developed by the World Bank to estimate the ambient concentration of PM_10_ on the city-level and used in the previous Global Burden of Disease (GBD) studies [71];
2. The global–regional chemistry transport model TM5, as well as the source receptor (SR) relationship, developed from TM5 which have been widely applied to evaluate the response of ambient air quality indicators to changes in emissions of various pollutants from the certain source in different control strategy scenarios [72,73,74];
3. Global atmospheric models such as GEOS-Chem [75] and MOZART [76], which use a similar approach, are also available to provide the ambient concentration estimates of ozone and/or PM_2.5_;
4. Land-use regression models which can estimate outdoor pollutant concentrations through specific geographic information of the source, landscape characteristics, and roadway [77,78];
5. Hierarchical Bayesian models are applicable for multiple-pollutants estimation by using tiered Bayesian statistical procedures [79,80].

### 2.3. Health Impact

The most important part of an AP-HRA is to quantify the health risk related to air pollution exposure. Various adverse health effects (also called health endpoints) attributed to short-term and long-term exposures can be categorized as follows:

1. For short-term exposure:
  - Mortality
  - Hospital admissions or emergency department visits caused by respiratory diseases
  - Hospital admissions or emergency department visits caused by cardiovascular diseases
  - Days of restricted activity
  - Absence from work or school
  - Other acute symptoms
2. For long-term exposure:
  - Mortality caused by cardiovascular and respiratory disease
  - Lung cancer
  - Chronic incidence caused by respiratory or cardiovascular disease
  - Decline in physiologic functions
  - Intrauterine growth restriction

Different subgroups of the population suffer the various risks of health effects caused by air pollution exposure. These vulnerable populations include ailing individuals, children and the aged, and sex differences would, in some cases, influence the level of burden of health effects as well.

Statistical data such as the mortality or morbidity rate among the population exposed to a particular air pollutant concentration is necessary. Numerous methodologies have been developed on short and long-term exposure (see Table 3), while most of them were conducted separately within different areas, resulting in generalizability limitation [67].

#### 2.3.1. Concentration-Response Functions (CRFs)

The health risk is represented by concentration-response functions (CRFs), which link the health endpoints attributed to exposure to air pollutant concentration changes. The relationship estimation between concentration change of air pollutants, ΔC and change in health effects (usually an incidence or mortality rate), Δy usually contains three steps: (i) determining a functional form of the CRF; (ii) estimating the coefficient values of the CRF; and (iii) deriving the relationship between ΔC and Δy from the CRF.

There are two forms for the CRF, linear and nonlinear. Linear and log-linear models are often used for simplification based on biological evidence [81,82,83,84], but nonlinear models (e.g., logistic model) may also be applied for comprehensive computation, depending on the baseline data, as well as specific air pollutants and endpoints [2]. For best regression fitness, the Akaike Information Criterion (AIC) approach may be used, and the model with a lower value of AIC is preferred [85]. Table 4 shows the different forms of CRFs which are widely used in health impact risk assessment studies.

In the above table, α represents a combination of all the independent variables, and β is the excess incidence rate of health outcome per 1 μg/m^3^ increase of pollutants.

#### 2.3.2. Relative Risk (RR)

The coefficient values of the CRF are typically derived based on Equation (1) from the level of Relative risk (RR), which describes the risk of an adverse health effect among the population exposed to a higher ambient air pollution level relative to a lower ambient level.

(1)  RR=exp(β×ΔC)

Previous epidemiological studies [12,86,87,88] postulated that RR associated with ambient air pollution is in a linear relationship with the concentration level, with several alternative linear function models established as below, where c represents the concentration of air pollutants and $c_{t}$ represents the minimum level below which there is no obvious adverse health impact (also called threshold value):

(2) $$\begin{array}{l} \mathrm{For}{\;c<c}_{t},\;{\mathrm{RR}}_{\mathrm{Lin}50}\left(c\right)=1,\\ \mathrm{For}\;c_{t}<c<50,\;{\mathrm{RR}}_{\mathrm{Lin}50}\left(c\right){=1+\gamma (c-c}_{t}),\\ \mathrm{For}\;c>50,\;{\mathrm{RR}}_{\mathrm{Lin}50}\left(c\right){=1+\gamma (50-c}_{t}). \end{array}$$

However, the studies focused on estimating the RR functions are mainly carried out in Europe and North America, where the pollutant concentration is low. Therefore, the models mentioned above may not be suitable for other regions, especially for developing countries where the concentration of the pollutant is relatively higher. Instead, the gradual diminution of the marginal increase in RR is extracted from the logarithm model [89] or power model [90,91] of RR and concentration. The WHO has subsequently recommended the logarithmic model for GBD to measure the health impact attributable to air pollution at the national level [92].

- Logarithm model:
  (3) $$\begin{array}{l} \mathrm{For}{\;c<c}_{t},\;{\mathrm{RR}}_{\mathrm{Log}}(c)=1,\\ \mathrm{For}{\;c\geq c}_{t},\;{\mathrm{RR}}_{\mathrm{Log}}(c)={\left[c+1/c_{t}+1\right]}^{\rho }. \end{array}$$

- Power model:
  (4) $$\begin{array}{l} \mathrm{For}{\;c<c}_{t},\;{\mathrm{RR}}_{\mathrm{Power}}(c)=1,\\ \mathrm{For}{\;c\geq c}_{t},\;{\mathrm{RR}}_{\mathrm{Power}}(c)=1+{\theta (c-c_{t})}^{\eta }. \end{array}$$

Based on the above mathematical forms used for burden assessment, recent studies have also conducted the meta-analysis of observed data and proposed an integrated exposure-response function (IERs) that flattens out at high exposures [93,94]:

(5) $$\begin{array}{l} \mathrm{For}{\;c<c}_{t},\;{\mathrm{RR}}_{\mathrm{IER}}(c)=1,\\ \mathrm{For}{\;c\geq c}_{t},\;{\mathrm{RR}}_{\mathrm{IER}}(c)=1+\alpha [1-{\exp(-\gamma (c-c_{t})}^{\delta })]. \end{array}$$

where α, γ, and δ jointly characterize the CRF which is derived from a fitting process.

#### 2.3.3. Result Integration

1. Mortality and morbidity:

Results of AP-HRAs are often summarized into several metrics, including numbers of deaths or diseases, years of life lost (YLL), disability-adjusted life years (DALY), or change in life expectancy [63].

The excess deaths or diseases (ED) derived from an increase in concentration can be calculated as follows:

(6) ED=Δy×Population

It can also be expressed in terms of the population attributable fraction [95,96,97]:

(7) ED=PAF×I×P

where PAF (population attributable fraction) is the fraction of disease burden attributable to pollution; I is the mortality incidence per year, and P is the all-age population. PAF can be then computed as below:

(8) $$PAF=\frac{p(\mathrm{RR}-1)}{p\left(\mathrm{RR}-1\right)+1}$$

where RR represents the relative risk of premature mortality obtained from the IER model, and p represents the fraction of the population exposed. When all people in the region of interest are exposed to the air pollutant, that is p=1.

2. Disability-Adjusted Life Year (DALY)

One DALY can be considered as one lost year of “healthy” life, while the total number of DALYs in the entire population can be regarded as the gap between an ideal health status where all people have no disease and disability and the current health status [98].

DALYs can be considered as the sum of YLL and YLD:

(9) DALY=YLL+YLD

YLL is a measure of the years of life lost due to premature death. The basic formula for a given cause, age, and sex is shown below:

(10) YLL=N×L

where N represents the number of deaths, and L represents standard life expectancy at the age of death in years.

YLD measures years lost due to disability. The basic formula considering the certain disease, age, and gender is shown below:

(11) YLD=I×DW×L

where I represents the number of cases, L represents the average years of disease, and DW represents the disability weight, reflecting the severity ranging from 0 (healthy) to 1 (dead).

### 2.4. Economic Assessment

The economic costs of the health effects can be monetized using two approaches: the value of a statistical life (VSL) method [99,100] and the cost of illness (COI) method [101].

VSL can be calculated through the willingness to pay (WTP) approach, which measures people’s willingness to pay for reducing a marginal death risk, following the equation shown as below [102]:

(12) $$\mathrm{VSL}=\frac{dWTP}{dP}$$

*WTP* represents the willingness to pay to avoid premature death and morbidity, and P represents the probability of death. The values of WTP are directly obtained through a survey-based conjoint analysis.

The cost of Illness (COI) method indicates the economic cost of some morbidity endpoints based on the mean estimation of unit values. Generally, the total COI comprises hospital admission cost, medical cost, and lost earnings due to missed workdays or restricted activity days. For this purpose, relevant data is obtained through the survey and interview with medical practitioners. Since the detailed information of treatment costs is not accessible in all regions, the following transfer approach can be used to calculate the illness treatment cost in the region i, in comparison with the European Union (EU) [103]:

(13) $$C_{morb(i)}{=C}_{morb(EU)}\times (\frac{{PCI}_{i}}{{PCI}_{EU}}{)}^{e}$$

where $C_{morb(i)}$ and $C_{morb(EU)}$ represent the illness treatment cost in the region i and EU country, ${PCI}_{i}$ and ${PCI}_{EU}$ are the per capita income in the region and EU, respectively. The value of $C_{morb(EU)}$ can be obtained from the European valuation table [104], and e is the elasticity coefficient of WTP [105].

## 3. AP-HRA Tools

There are currently various quantitative HRA tools developed by governmental and non-governmental entities to provide timely information regarding air pollutant exposure and its health impacts. Among them, COBRA (Co-Benefits Risk Assessment), Simair, Air Q+, BenMAP-CE (Environmental Benefits Mapping and Analysis Program—Community Edition), Ecosense, Household Air Pollution Intervention Tool (HAPIT), GAINS (Greenhouse gas—Air pollution Interactions and Synergies model) were developed to quantify the number of air pollution-related premature mortalities, disability-adjusted life years, and cases of disease [106]. These tools use common data for population, sources for baseline mortality rates, and concentration-response associations, but they vary in degree of technical complexity, exposure information source, and format [107]. They use a different methodological approach, spatial resolution, and geographical scope. However, most of these tools are preset to estimate the effects of NOx, Sulfur Oxides (SOx), PM_2.5_, and PM_10_. The input data can also vary depending upon the source of air pollution and its impact on a specific population or sub-population like children or air pollution by a particular sector [52,108]. Some of the tools allow user-specified inputs. However, most of these tools use default values for demographic, concentration-response functions, and health data to estimate the population’s exposure level. Table 5 represents some of the widely used quantitative HRA tools.

BenMap-CE estimates health impacts and monetary benefits from reductions in PM_2.5_ and ozone. The possible economic consequences of air pollution-related health impacts can be quantified by BenMap-CE, enabling users to measure the potential health and economic benefits of improving air quality in any country or region of the world, using the air quality, population, baseline health, and concentration-response criteria of the GBD. [120]. The health impacts include heart attacks, Premature mortality, and other air pollution-related health effects due to air quality changes. After determining ambient air quality changes using user-specific air quality data, BenMAP-CE relates health effects or health endpoints with changes in the air pollution concentration, using CFRs.

HAPIT is a web-based tool that was developed to estimate the expected health benefits from low indoor PM_2.5_ emission development strategies in middle and low-income countries. It can be used to estimate averted premature deaths and DALYs and health-associated costs of the different intervention scenarios by using the best available background disease and data available for the exposure-response [133]. HAPIT can be used to evaluate the implication of the intervention scenarios for improving indoor air quality in countries where a significant portion of the population uses solid fuel, allowing policymakers to compare the relative merits of interventions within and between different countries. HAPIT depends on up-to-date national health background information and the tools and databases built for the Comparative Risk Assessment (CRA) which were used for the 2010 Global Burden of Disease (GBD 2010). Exposure-response details are used in 57 countries where solid fuels account for 50% of primary cooking fuel [134].

COBRA evaluates the human health and economic impacts of the state-level low emissions development strategies in the US by translating the reduced PM and other concentrations of air pollutants into preventable causes of death. It helps identify the best option with the highest health benefits or reduce health risks in a cost-efficient manner [135]. COBRA uses county-level predicted PM_2.5_ concentrations as a proxy of PM_2.5_ exposures for individuals living in those counties and estimates the health effects by comparing them with exposure-response relationships based on the available data from the EPA. A Gaussian dispersion model is being used in the COBRA tool that accounts for dry and wet deposition as well as first-order chemical atmospheric transition. The S-R matrix includes transfer coefficients in the U.S. between emissions and county-level PM_2.5_ concentrations and integrates meteorological inputs determined in the 1990 EPA guideline impact analysis based on weather observation [136].

The Simple Interactive Model for better Air quality (SIM-air) is used to assess the implications of the integrated air quality management policies in developing countries’ urban areas. It combines the Geographical Information System (GIS) with the local emission data inventories in cities in evaluating various air quality scenarios. SIM-air uses the source-receptor transfer matrix (SRTM) to convert emissions of the concentrations, which is an output from a chemical transport model. It provides the necessary information for the policymakers to prioritize their air quality management policies, optimizing options for both public health and costs impacts in order to better adapt to local ambient standards in urban areas [53,137].

AirQ+ software tool for health risk assessment of air pollution is one of the most widely used tools for calculating the possible health impacts of improving air quality. It assesses the short-term and long-term exposure to both outdoor and indoor emissions of PM_10_, PM_2.5_, O_3_, NO_2_, and black carbon. AirQ+ helps measure the health impacts of atmospheric and household air pollution and aims to measure cancer risks and contain unit risk values for nickel, benzene, vinyl chloride, and chromium (VI) arsenic, and benzopyrene calculates the number of preventable premature deaths and diseases due to improvement in the air quality using the Health Impact Function (HIF) equations. The HIF estimates the count of premature deaths and diseases by using baseline rates of mortality or morbidity, population data, air pollutant concentrations, concentration-response parameters [120]. EcoSense is an atmospheric dispersion and air pollution exposure assessment model that helps estimate the health and environmental impacts and related economic impacts in Europe. It calculates long-term effects on human health, ecosystem, and crops by airborne pollutants, taking into account the chemical transformational and dispersion of pollutants. The CRFs are used to quantify the DALYs and morbidity rates causes by long-term exposure to NO_2_, PM, and Ozone [138,139]. EcoSense integrates local and regional dispersion models with complex exposure-response network functions to quantify the impacts of elevated concentrations of air pollutants and also the economic value for the different impact categories like human health, building materials, forests and ecosystems, and crops.

GAINS model identifies the cost-effective portfolios of pollution reduction policies that achieve air quality improvements at a minimum cost. GAINS helps address the risks of fine particulate matter and ground-level ozone to human health and the danger of acidification disruption to habitats, excess nitrogen accumulation (eutrophication), and exposure to high ozone levels. The environmental and health impacts of primary pollutants (PM_2.5_-PM_10_) particles, sulfur dioxide (SO_2_), non-methane volatile organic compounds (VOC), ammonia (NH_3_), and nitrogen oxides (NOx) are quantified in a multi-pollutant context. For the change in the emissions, source-receptor relationships have been established, and compressive transport models together with the atmospheric chemistry are used to simulate complex physical and chemical reactions [140]. The GAINS uses the Eulerian Unified EMEP model for assessment describing the fate of atmospheric pollutants [141]. Health impact estimation of GAINS is based on epidemiological studies quantifying mortalities due to the long-term exposure to PM_2.5_ or SOMO35.

Table 6 represents the comparison between the above-mentioned AP-HRA tools, concerning their methodologies, scopes, input parameters, and predicted health impacts. 

## 4. Discussions

Air pollution health risk assessment tools have different advantages when it comes to simplicity, consistency comparability, and quality assurance. These tools also help policymakers by providing necessary information to make action plans to reduce air pollutants by reducing the combustion of fossil fuels. Substantial progress has been made in evaluating the health and other environmental effects of the HIA tools. The number of these tools has advanced over the past decade because of growing epidemiological data that offers quantitative parameters of air emissions and health impact the concentration-response relationship, which has helped decision-makers educate the public about the potential estimated benefits of improved air quality [142]. Simultaneously, low-quality baseline morbidity rates, especially in low-income countries, make it challenging to measure air-pollution-related morbidity effects worldwide [107] accurately. Each of these tools has its limitations and strengths. Knowing them is crucial while assessing the health and economic impact of air pollution. A comparative SWOT (strengths, weaknesses, opportunities, and threats) analysis of the tools mentioned above has been carried out in this research, which is summarized in Table 7.

To estimate air pollution, most tools rely on air quality modeling, but some may also collect these data from air quality monitor observations or derive information from both monitors and models. Using the models for health impact assessment offers an advantage to cover a broader spatial area. On the other hand, monitoring data represents real atmospheric concentrations over a discrete amount of time in a given area [107].

There are several complexities in the use of air quality models for health impact assessment. In epidemiological studies from which concentration-response comparisons are extracted, modeled concentrations do not correlate to the method or spatial resolution of the characterization of exposure and may contribute to the inaccuracy of the analysis. In addition to that, the inherent uncertainty of simulated concentrations may not have enough resolution to represent the actual patterns of exposure. So, it sometimes becomes a challenge to deliver reasonable outcomes for policymakers and other people who do not have specialized skills in the field while keeping harmony between tools utilized and the multifaceted nature of the data.

It is essential to use the most precise and highly accurate data in the health impact assessment tools [144]. In addition to that, some unknown uncertainties and their interaction with each other are also usually not known. Like the air we breathe could blend different pollutants with various sources and pass through different chemical reactions in the atmosphere. Furthermore, considering air pollution as the only factor responsible for many health outcomes and mortalities may not be the only solution. There are multiple factors, such as social and cultural behaviors, and should be considered in AP-HRA tools [144]. While developing a tool for HIA studies, the main features like spatial resolution, emissions, health impacts, population exposure characterization methods, accessibility, sophistication, and application in policy contexts should be considered.

## 5. Conclusions

This study presents the scope and importance of air quality health risk assessment (AP-HRA) and outlines the methodological approaches. AP-HRAs contain the estimation and modeling of processes including population estimates, population exposure to air pollutants, adverse health impacts assessment, and economic assessment, among which the health impact assessment is the core part, with specified concentration-response functions and relative risks for different cases of interest as the most significant methodological models and parameters for quantification. In addition, in this paper, we reviewed seven widely used air pollution health impact assessment tools. These tools, usually designed for a specific assessment context, vary in geographical scope, resolution, method approach, technical quality, and alternative aspects. Furthermore, nearly all of these tools use similar knowledge sources for population, baseline mortality rates, and concentration-response associations. Many of the tools mentioned in this paper have played a leading role in highlighting the health and economic impacts of low air quality and have directly contributed to environmental initiatives to increase air quality. Those conducting AP-HRA need to know what data are available and the way to communicate the results. When selecting the tool, it is essential to define first the technical needs of the assessment, the geographic scale, and the relevant pollutants. In addition to that, while selecting a study location, potential differences in exposure patterns, pollution characteristics, lifestyle, population behavior, and medical care system should also be considered.

Future work concerns the in-depth comparative analysis of particular AP-HRA tools, mainly COBRA and GAINS, to quantify multiple (heath, environmental, and economic) impacts of the clean transport scenario in Delhi, India, and de-capacity potential and clean energy policies in the industrial sectors in the selected provinces in China. The objective would be to gain a better understanding of the similarities and differences in the approaches used in these tools to achieve the operationalization of HIA in the selected regions.

## Figures and Tables

**Table 1:** Recent studies in the air pollution health risk assessment.

Purpose of the Study	Region	Health Impacts	Ref
Evaluating the mortality impact of fine particles reduction policies and Air quality modeling in Spain.	Spain	All-cause deaths	[48]
Assessing the geographical spread and economic benefit of the ozone health consequences associated with climate change in the United States in 2030	USA	Mortality and morbidity impacts related to ozone	[49]
Reductions of PM_2.5_ Air Concentrations and Premature Mortality in Japan	Japan	Mortality	[50]
Assessing the health-related benefits of attaining the ozone level standard	USA	Mortalities, emergency department admissions, hospitalization, restricted activity day, and school absences	[51]
Estimation of the national public health burden associated with exposure to atmospheric PM_2.5_ and ozone	USA	Reduced life years and life expectancy; and mortalities	[52]
Evaluation of air quality in six Indian cities to create a knowledge base for multi-pollutant pollution, dispersion modeling of ambient particulate concentrations	India	Premature mortality	[53]
Evaluation of the health-related economic externalities of air emissions from particular emission sources or industries that can be used to help emission reduction policy-making.	Europe	Mortality and morbidity	[54]
Using multi-sectoral emissions inventory to estimate health impacts in terms of premature mortality and morbidity in Delhi	Delhi, India	Premature mortality and morbidity effects	[55]
Health benefits from the adaptation of cleaner brick processing technologies	Dhaka, Bangladesh,	Mortality and morbidity, health cost savings	[56]
Study the linkages between indoor and outdoor PM in Ulaanbaatar, Mongolia	Ulaanbaatar, Mongolia	Premature deaths	[57]
Estimation of the citywide morbidity and mortality attributable to ambient fine particulate matter (PM_2.5_) and ozone in New York City	New York City, USA	Health impacts and disparities	[35]
Assessment of the intercontinental impact of ozone emissions on human mortality	Northern Hemisphere, North America, East Asia, South Asia, and Europe	Premature mortality	[58]
Estimation of the mortality impacts of 20% of anthropogenic primary PM_2.5_ and PM_2.5_ precursor emission decreases in each of the four major industrial regions (North America, Europe, East Asia, and South Asia)	Europe, East Asia, and South Asia, North America,	Premature mortality	[59]
Evaluation of the external health costs of air emissions in Europe and the contribution of international shipping activities	Europe	Health-related cost of Air pollution	[54]
Calculation of premature deaths from cardiopulmonary and lung cancer due to PM_2.5_ levels and the effect of reductions in black carbon emissions on surface air quality and human mortality	Global	Mortality	[60]
Estimation of premature air pollution-related mortalities prevented, ozone-related yield reductions of large food crops avoided and health damage avoided	Global	Mortalities, Morbidities and avoided Ozone-related reduction of yield of major food crops.	[61]
Estimating the global and national health burden of atmospheric PM_2.5_ pollution due to surface transport emissions.	Global	Mortality	[62]

**Table 2:** Air quality indicators of typical air pollutants.

Pollutant	Indicator		Interim Target-1	Interim Target-2	Interim Target-3	Air Quality Guideline (AQG)
PM_2.5_	annual mean	10 μg/m^3^	35	25	15	10
	24-h mean	25 μg/m^3^	75	50	37.5	25
PM_10_	annual mean	20 μg/m^3^	70	50	30	20
	24-h mean	50 μg/m^3^	150	100	75	50
O_3_	8-h mean	100 μg/m^3^_-_	160	-	-	100
NO_2_	annual mean	40 μg/m^3^	-	-	-	-
	1-h mean	200 μg/m^3^	-	-	-	-
SO_2_	24-h mean	20 μg/m^3^	125	50	-	20
	10-min mean	500 μg/m^3^	-	-	-	500

**Table 3:** Epidemiological studies of short- and long-term exposure and their features.

Category	Methodology	Advantage	Disadvantages
Short-term exposure	Time-series studies: using the statistical model to estimate the influence of temporal (usually daily) changes of air pollutant concentrations on daily health incidence in the population exposed.	Avoid disturbance caused by long-term variations such as individual occupations and socioeconomic conditions; lower costs associated with data collection.	Uncertainty caused by the quality of health data; Unable to quantify the chronic effects of air pollutants.
	Case-crossover studies: studying the risk of an acute health case after momentary exposure.	Get rid of confounder from time-independent factors; Improve causal inferences on the individual level.	Unsuitable to estimate the risk from exposures with a time trend.
	Panel studies: assessing the respiratory diseases associated with air pollution among susceptible subgroups.	Availability of detailed health- and exposure-related information of individuals.	Uncertainty caused by the relatively small sample size.
Long-term exposure	Cohort studies: examining the risk of health endpoints attributed to long-term pollution exposure.	Consider the total impact of all types of health cases.	High cost and complication of implementation; High demand for spatial, temporal and average concentration data.

**Table 4:** CFRs in health impact risk assessment.

Functional Form	Formula of CRFs	Relationship between ΔC and Δy
Linear function	y=α+β×C	$\Delta {y=y}_{0}{-y}_{c}=\beta \times \left(C_{0}-C\right)=\beta \times \Delta C$
Log-linear function	ln(y)=α+β×C	$\Delta {y=y}_{0}{-y}_{c}{=y}_{0}(1-\frac{1}{\exp(\beta \times \Delta C)})$
Logistic function	$y=\mathrm{prob}(\mathrm{occurrence}\;|C\times \beta )=(\frac{\exp(C\cdot \beta )}{1-\exp(C\cdot \beta )})$	

**Table 5:** Widely used quantitative HRA tools.

Tool	Developer	Study Area	Reference
Environmental Benefits Mapping and Analysis Program—Community Edition (BenMap-CE)	The United States Environmental Protection Agency (EPA)	USA, Turkey, Spain	[46,48,109,110]
Greenhouse gas—Air pollution Interactions and Synergies (GAINS) model	International Institute for Applied Systems Analysis (IIASA)	Europe	[47,111,112]
CO-Benefits Risk Assessment (COBRA) Health Impacts Screening and Mapping Tool	The United States Environmental Protection Agency (EPA)	USA	[113,114,115]
Air Quality (Air Q+)	World Health Organization (WHO)	Iran, Italy	[116,117,118,119]
Air Q+ and BenMAP-CE	EPA and WHO	USA	[120]
The Simple Interactive Model for better Air quality (SIM-air)	Urban Emissions	India, Europe	[53,121,122]
Household Air Pollution Intervention Tool (HAPIT)	Household Energy, Climate, and Health Research Group at the University of California, Berkeley	India	[123,124,125]
Ecosense	Institute of Energy Economics and Rational Energy Use (IER), University of Stuttgart	Greece France, Brazil	[126,127,128]
TM5- FASST	JRC Ispra (Italy)	China, Multinational study	[30,129]
Aphekom	French Institute of Public Health Surveillance	25 European cities, 10 European cities	[130,131,132]

**Table 6:** Comparison between the AP-HRA tools.

Characteristic	AIRQ2.2	BenMAP-CE	COBRA	HAPIT	SIM-Air	GAINS	EcoSense
**Health Impacts**							
Mortality (cases)	**√**	**√**	**√**	**√**	**√**	**√**	**√**
Disability-adjusted life years (DALY)	**√**	**√**	**√**	**√**		**√**	**√**
Morbidity (cases)	**√**	**√**	**√**	**√**	**√**		**√**
Economic Impacts	**√**	**√**	**√**		**√**		**√**
**Pollutants:**							
PM_2.5_	**√**	**√**	**√**	**√**	**√**	**√**	**√**
PM_10_	**√**	**√**			**√**	**√**	**√**
Ozone	**√**	**√**				**√**	**√**
NO_2_	**√**	**√**	**√**			**√**	**√**
SO_2_	**√**	**√**	**√**			**√**	**√**
CO	**√**	**√**				**√**	**√**
Other	Black smoke		VOC			CO_2_, VOC, CH_4_, N_2_O	Hydrocarbons, dioxins and heavy metals
**Spatial Resolution**							
Regional	**√**	**√**			**√**	**√**	**√**
National	**√**	**√**	**√**			**√**	
City-level	**√**	**√**			**√**	**√**	
Household/Indoor	**√**			**√**		**√**	**√**

**Table 7:** SWOT (strengths, weaknesses, opportunities, and threats) analysis of the selected AP-AHP tools.

Tool	Strength	Weakness	Opportunities	Threats
**AirQ+**	- Health impacts Quantification of indoor/outdoor air pollution. - Quantification of the cancer risks and includes unit risk values for chromium (VI), arsenic, nickel, benzene, vinyl chloride, and benzopyrene is an additional feature in the tool. - Multilanguage versions of the tool are available.	Evidence-based health outcome relationships are not strong, especially with the air pollutants like NO_2_, BC (Black Carbon), and long-term ozone exposure.	There is an opportunity to refine further the spatial resolution in the analysis carried out with AirQ+ and integrate new user-friendly features like additional explanations for input data and components to calculate economic impacts and DALYs.	Often unrefined spatial resolution in the analysis is carried out with AirQ+, which may cover a whole country or city’s spatial domain [120].
**COBRA**	- It helps researchers create a new scenario that suggests improvements in pollution from baseline emissions smoothly and efficiently. - Detailed and comprehensive estimation of the health and economic gains that are related to decreasing the atmospheric PM2.5 concentrations over a given year of study.	- Entirely concentrated on state-wise health impacts assessment in the US, making it difficult to be used in other regions. - The SR Matrix does not reflect the interaction which takes place in the atmosphere between the air pollutants.	Currently, COBRA has baseline data, which is only appropriate for the USA. There is an opportunity to add baseline data to make it suitable for regional or global HIA studies. The tool needs to continue to evolve and integrate the functionality and improve the sophistication of analysis.	- Some health endpoints like, upper respiratory symptoms, lower respiratory symptoms, and acute bronchitis are using a comparatively small sampling group and estimated from a single local survey, which increases the estimation’s uncertainty.-For consistent distribution of air pollutants, an initial probabilistic method adjusted by the developers has been only used in the COBRA, which reduces the accuracy of the results.
BenMAP—CE	Merging the CFRs with basic pooling strategies (e.g., random effects and fixed effects) to construct a new function that can adequately consider the diverse demographics data.	- The degree to which different mixtures of air pollutants pose a greater or lesser risk and the extent to which concentration-response associations observed in one group is limited to the particular case studies and cannot easily be extended to other cases. - Estimating health impacts due to air quality is limited to a single year period and cannot be carried out on a multiple-year horizon [107].	Incorporating new features into the tool, such as the estimate of the health impacts due to the exposure to multiple pollutants [120].	Spatial shifts in city-wide environmental concentrations, diverse sets of individual activity patterns, and indoor ambient air pollution differences [142].
**HAPIT**	- HAPIT is an easy-to-use tool that helps estimate averted DALYs, averted premature deaths, and choosing Cost-Effective interventions. - Information on total households studied in the intervention, PM2.5 exposure to pre and post-intervention population, and the average proportion of the population using intervention helps estimate the cost per intervention of the initiative the annual operating costs per household.	- The estimation period is short cannot be indicative of long-term trends. - Equal exposures among household members is assumed in the HAPIT. However, the exposure levels vary among the household members.	To decrease the uncertainty in the results, information about the baseline and intervention PM_2.5_ exposure levels should be included for the developing countries where solid fuel is mostly used.	Background diseases and economic characteristics of a population are assumed to remain relatively unchanged in HAPIT. This presumption will hold for a short life-span. Therefore, for long-term interventions, such as shifting from fossil fuel to renewable energy or electricity, the forecasts will have to be periodically updated.
GAINS	Compressive Transport models and atmospheric chemistry to simulate complex physical and chemical reactions [140].	- The atmospheric dispersion model in GAINS is simplified into the basic linear function form based on the regression of results from TM5 and the relevant response-source model, resulting in uncertainty. - The health impact is assessed according to general RR value obtained from European and American epidemiological studies, which is unsuitable and inaccurate for other areas [140].	- Future projections of activity data such as macroeconomic drivers, energy, and fuel consumption are exogenous to the GAINS model, derived from other model calculations or national experts provided to ensure timeliness and authority. - Alternative pathways can also be specified in the GAINS Expert mode, improving the applicability for more scenarios.	Other models that focus on emission estimation or health impact assessment separately can provide more precise results and, if combined, would be a better alternative option than GAINS.
**ECOSENSE**	- Comprehensive estimation of air pollution impacts on human health and Ecosystems. - Robust database including details of major air pollutants, hydrocarbons, and heavy metals [143].	Considering a simple linear source-receptor model for assessing the atmospheric chemistry interactions that perform a nonlinear behavior in nature [107,128]).	Validation of the meteorological models used in the EcoSense tool to make it more appropriate for the developing countries by reviewing the meteorological databases and concentration-response functions.	- Inability to capture complicated atmospheric chemistry processes [107]. - The exact estimation of the form and severity of the related environmental impacts is hindered by limited knowledge of receptor size [126]. - Present projections of the external cost of climate change vary considerably, reflecting the high uncertainty of the forecasts since much of them would take place over the long term.
**SIM-AIR**	Multiple benefits (Environmental—health—economic) assessment of the climate change action plans, considering interactions between emissions, dispersion of pollution, impacts, and options for management [53,137].	Uncertainty in spatial analysis resolution matching the project (mainly urban areas).	For the study of pollution inventories and health effects, the database of concentration-response functions and emission sources is included in the tools that can be modified with relevant data from cities.	Recognizing the uncertainty of inventories is important and needs to be adjusted carefully as per the local data.

## Data Availability

Not applicable.
